# Optimization, validation and initial clinical implications of a Luminex-based immunoassay for the quantification of Fragile X Protein from dried blood spots

**DOI:** 10.1038/s41598-022-09633-8

**Published:** 2022-04-04

**Authors:** Anna E. Boggs, Lauren M. Schmitt, Richard D. McLane, Tatyana Adayev, Giuseppe LaFauci, Paul S. Horn, Kelli C. Dominick, Christina Gross, Craig A. Erickson

**Affiliations:** 1 Division of Child and Adolescent Psychiatry (MLC 4002), Cincinnati Children’s Hospital Medical Center, 3333 Burnet Avenue, Cincinnati, OH 45229-3039 USA; 2 Division of Developmental and Behavior Pediatrics, Cincinnati Children’s Hospital Medical Center, 3333 Burnet Ave., Cincinnati, OH 45229-3039 USA; 3 Department of Pediatrics, University of Cincinnati College of Medicine, Cincinnati, OH USA; 4 Department of Psychiatry and Behavioral Neuroscience, University of Cincinnati College of Medicine, Cincinnati, OH USA; 5 Division of Neurology, Cincinnati Children’s Hospital Medical Center, 3333 Burnet Ave., Cincinnati, OH 45229-3039 USA; 6 Department of Human Genetics, New York State Institute for Basic Research in Developmental Disabilities, Staten Island, NY 10314 USA

**Keywords:** Molecular biology, Neuroscience

## Abstract

Fragile X Syndrome (FXS) is caused by a trinucleotide expansion leading to silencing of the *FMR1* gene and lack of expression of Fragile X Protein (FXP, formerly known as Fragile X Mental Retardation Protein, FMRP). Phenotypic presentation of FXS is highly variable, and the lack of reproducible, sensitive assays to detect FXP makes evaluation of peripheral FXP as a source of clinical variability challenging. We optimized a Luminex-based assay to detect FXP in dried blot spots for increased reproducibility and sensitivity by improving reagent concentrations and buffer conditions. The optimized assay was used to quantify FXP in 187 individuals. We show that the optimized assay is highly reproducible and detects a wide range of FXP levels. Mosaic individuals had, on average, higher FXP levels than fully methylated individuals, and trace amounts of FXP were consistently detectable in a subset of individuals with full mutation FXS. IQ scores were positively correlated with FXP levels in males and females with full mutation FXS demonstrating the clinical utility of this method. Our data suggest trace amounts of FXP detectable in dried blood spots of individuals with FXS could be clinically relevant and may be used to stratify individuals with FXS for optimized treatment.

## Introduction

Fragile X Syndrome (FXS) is the most common single gene cause of autism spectrum disorder (ASD) and most common inherited cause of intellectual disability impacting 1 in 4000 males and 1 in 6–8000 females worldwide^1^. FXS results from CGG triplet repeat expansion in the promotor region of the *FMR1* gene located on the long arm of the X chromosome^2^. Typically, over 200 CGG repeats result in gene methylation and transcriptional silencing of the *FMR1* gene. The CGG repeats in the full mutation range are usually inherited and undergo expansion when passed from a premutation carrier (PMC) mother (55–200 CGG repeats) to her child^2^. As a disorder of gene silencing, FXS results from deficient production of the *FMR1* gene product, Fragile X Protein [FXP, previously termed fragile X mental retardation protein (FMRP)]^3^. FXP has many functions, including serving as a translational repressor impacting the expression of hundreds of proteins vital to brain function, including those critical to cognitive functioning^4^.

FXS is associated with a behavioral phenotype marked by high incidence of anxiety, ADHD, language and cognitive deficits among other clinical features^5–7^, and with physical presentations including, but not limited to, pronounced ears, soft tissue laxity and macroorchidism in males^8^. Despite commonalities in the presentation, significant variation in the behavioral phenotype does exist within FXS. Females with FXS are obligate mosaics with two X chromosomes resulting in a highly variable phenotype in girls and women ranging from no appreciable developmental impairment to significant development delay or intellectual disability. Even among males with FXS, phenotypic developmental variability is represented by functioning levels. from severe to mild, or even borderline intellectual/cognitive impairment^8^. In part, this variation may be due to mosaicism in FXS. Repeat size mosaicism can occur when individuals have a mix of premutation and full mutation repeat alleles, whereas methylation mosaicism can occur where clinical Southern Blot (SB) and Polymerase Chain Reaction (PCR) testing may indicate inconsistent methylation patterns with a mix of fully and non-fully methylated *FMR1* alleles regardless of CGG repeat length. Given the phenotypic and genetic variation in FXS, it is of critical importance to understand how this variation may relate to variable FXP expression in this disorder.

Evaluation of FXP expression in individuals with FXS has been challenging due to difficulties with sensitivity and specificity of available assays. In addition, there are limited studies assessing how well FXP levels in accessible peripheral cells reflect FXP levels in the brain and whether FXP in peripheral cells is associated with characteristic phenotypic features. Nevertheless, in recent years there has been considerable progress in methodology for FXP detection and measurement in peripheral tissue, such as human blood, skin fibroblasts, hair follicles, and buccal cells^9–14^. One of the first attempts to evaluate FXP levels used immunofluorescent staining in blood lymphocytes^15^. This method was subsequently used to document the significant relationship between higher FXP levels and higher cognitive functioning based on IQ scores^6,16,17^. However, this assay did not take into account different expression levels of FXP in individual lymphocytes and thus lacked sensitivity to detect a spectrum of FXP expression, which may be clinically relevant. Despite western blot and enzyme-linked immunosorbent assay (ELISA) methods providing a continuous readout that better captures the full range of FXP expression, these methods are difficult to scale up (western blot) and lack quantifiability, sensitivity and/or specificity (reviewed in^14^).

Recently, a novel, highly sensitive assay was developed measuring FXP as a continuous variable in peripheral blood using Luminex-based technology^18,19^. This method is not only more sensitive to detect lower values of FXP than previous methods, but the Luminex-based assay is easily scalable, requires less sample volume, and has increased specificity due to the use of two different highly specific FXP antibodies. Moreover, this assay can be used with eluates from dried blood spots, which facilitates the potential future application of this assay outside the research laboratory and into clinical settings. The FXP Luminex-based immunoassay was used in a recent study to measure FXP levels in 42 samples of individuals with FXS and demonstrated that males with severe intellectual disability (ID) had lower FXP than males with mild or moderate ID^20^. However, the lower limit of FXP detection was above zero indicating difficulty differentiating “true zero” FXP expression from potential low or trace level FXP expression, thus also limiting its capacity to be more clinically relevant among males with full mutation (FM) FXS.

Thus, given the major advantages the Luminex-based immunoassay offered over previous methods to measure FXP, we sought to optimize this method to improve upon the detection of FXP in peripheral blood to best discern potential molecular variation in FXS. We believe a highly sensitive and reproducible FXP assay will be important to new treatment development as FXP expression likely varies extensively in FXS despite the single gene nature of the disorder. In appreciating molecular variation, we aim to, in the future, use this understanding as a means to biologically subgroup persons with FXS when evaluating clinical presentation, outcome, and response to potential therapeutics. Here, we report on our initial work to optimize FXP detection in human blood with a focus on enhancing assay accuracy and improving the lower limit of detection using the FXP assay in broad subgroups of individuals, with emphasis on populations of males and females with FM FXS.

## Methods

### Participants

We enrolled a total of 187 participants: 101 males and 86 females aged 0–78 years (Table 1). Participants with a FM (70 males and 33 females) or premutation (7 males and 42 females) in the *FMR1* gene were recruited through the Cincinnati Fragile X Research and Treatment Center. Fragile X status was confirmed at minimum by clinical SB and/or PCR testing to confirm group assignment. Among individuals with FM FXS, 54 males and 29 females had reliable research standard SB and PCR analysis completed at Rush University to evaluate for repeat size mosaicism, methylation mosaicism, or expression of both mosaicisms. Within this subsample, 18 males (33.3%) exhibited methylation and/or size mosaicism and 9 females (31%) had methylation mosaicism in addition to being obligate size mosaics. Typically developing control (TDC) subjects (24 males and 7 females) were recruited through web-based fliers from the local community and had no prior diagnosis or treatment for developmental or neuropsychiatric disorders. All participants or their legal guardians gave written informed consent and/or verbal assent, when appropriate. This project was approved by the CCHMC Institutional Review Board. The human subjects work was completed in accordance with all relevant guidelines and regulations, including being in accordance with the Declaration of Helsinki.

**Table 1:** Demographic information.

	No	Age			FXP concentration (pM)			
		Average	SD	Range	Average	Median	SD	Range
**Male**	**101**	**19.2**	**15.3**	**0.3**–**61.6**	**10.2**	**1.7**	**13.4**	**0.0–43.2**
Typically developing controls	24	26.0	14.9	5.5–60.1	28.9	27.5	6.4	18.1–43.2
Premutation carriers	7	31.1	23.4	2.2–61.6	30.5	26.1	7.9	22.6–42.1
Fragile X Syndrome	70	15.7	13.2	0.3–45.7	1.7	0.5	2.6	0.0–10.3
Nonmosaic	36	18.2	14.6	0.3–45.7	0.6	0.3	1.2	0.0–6.2
Mosaic	18	14.8	13.8	0.5–41.5	3.8	2.6	3.2	0.0–10.3
**Female**	**86**	**33.2**	**18.9**	**0.2–78.4**	**26.4**	**24.8**	**10.6**	**3.1–64.9**
Typically developing controls	11	20.9	18.9	0.2–63.8	31.6	27.5	13.7	16.1–64.9
Premutation carriers	42	47.3	13.2	9.0–78.4	30.1	28.5	9.6	11.8–55.2
Fragile X Syndrome	33	19.4	10.3	1.2–42.9	19.9	20.9	7.1	3.1–33.8
Methylation mosaicism	9	16.4	7.8	1.2–25.2	22.0	20.9	7.7	9.3–30.6

*FXP* Fragile X Protein, *SD* Standard deviation.

### Blood collection and processing

**Figure 1:**
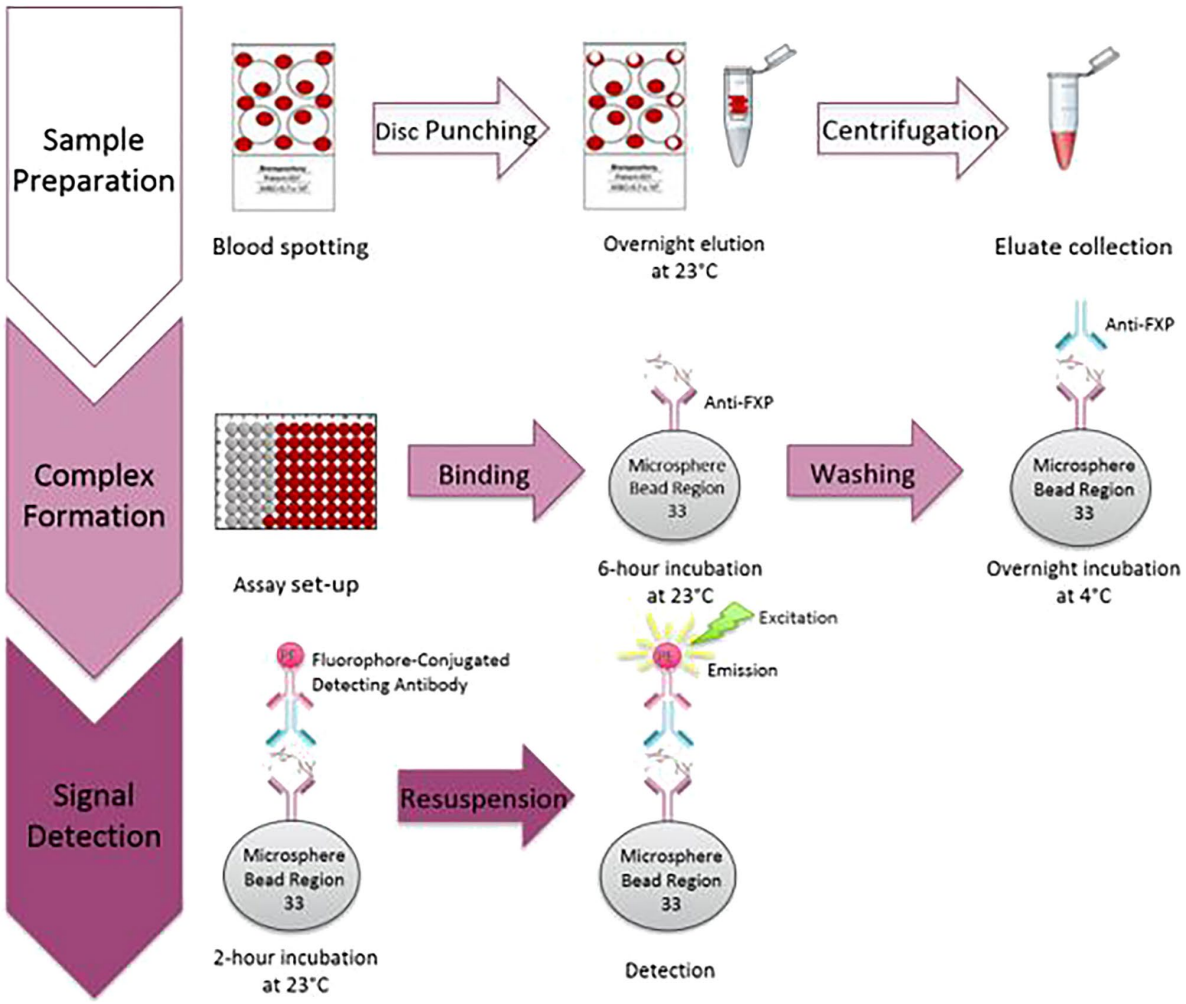
Experimental workflow of the optimized FXP assay. *FXP* Fragile X Protein.

Blood samples were collected from all participants in 2 mL Vacutainer K2EDTA tubes (BD, 36781) and inverted 10 times before processing to ensure homogeneity within the sample. Fifty microliters of blood were pipetted onto Bloodstain Cards (Whatman Bloodstain Cards, WB100014) producing at least two cards with 13 spots each from one sample collection. Cards were dried and stored with desiccant packs in low-gas-permeable bags (VWR, 89027-022) within 4–24 h after spotting to ensure DBS stability, in accordance with dried blood spot (DBS) guidelines and published protocols^21,22^ (Fig. 1). DBS cards were either used immediately after drying or stored at – 80 °C for a maximum of 2 years before analysis.

### Elution of DBS

From each card, five 6.9 mm diameter disks were prepared using a hole punch and transferred into CoStar Spin-X Centrifuge Filter Tubes (7200388). Proteins were extracted from the DBS using 333 µL of elution buffer (M-PER with salt, Antipain, Chymostatin, Protease Inhibitor) with orbital shaking overnight at room temperature. The eluates were collected after a 6-min centrifugation at 12,000×*g* and immediately used in the assay. Fifty microliters of the eluate were used per well in the assay (Fig. 1).

### Immunoassay procedure

An 11-point standard curve was created using a purified GST-SR7 fusion protein obtained from the Institute for Basic Research in Developmental Disabilities (IBR). Elution buffer was used to complete a two-fold dilution of the first standard point generating a standard curve with range of 0.07–70 pM. Either 50 µL of standard protein or DBS extract was aliquoted into assay wells of a 96-well low protein binding plates (Greiner Bio-One, 655096). The capture antibody, mAb 6B8 (BioLegend, 834601), was concentrated according to manufacturer’s instructions (Abcam, ab102778). The concentrated mAb 6B8 was coupled to Luminex Magspheres (Luminex, MC10033-01) according to manufacturer’s instructions and constructed at a stock concentration of 100µL antibody/12.5 million beads. Beads were diluted in assay buffer (PBS, 1% BSA, 0.05% Tween) to 80 beads/µL for use in assay. Diluted beads were added to the assay wells at a volume of 50 µL to bring the final well volume to 100 µL. Plates were then incubated at room temperature for 6 h on a microplate shaker. A Luminex magnetic plate separator was used to manually wash the plates in assay buffer. After washing, the plates were incubated overnight in secondary detecting antibody (ab17722, Abcam) at a dilution of 1:1000 (v:v). Plates were vigorously washed then incubated at room temperature for 2 h in signal detecting antibody (Jackson ImmunoResearch, 711-116-152). Plates were vigorously washed and resuspended in 100 µL of sheath fluid (Luminex, 40-50021). The magspheres were analyzed (in quintuplicate) on the Luminex 200 system utilizing XPonent Software (Version 4.2) to determine median fluorescence intensity (Fig. 1).

### Fragile X Protein (FXP) quantification

To determine individual FXP concentration, BioPlex Manager Software (Version 6.2) was used to generate a standard curve of GST-SR7 concentration as a function of median fluorescence intensity. Patient samples were plotted against this curve and reported as concentration (pM) in the DBS extract. Based on the volume of blood spotted, the size of the DBS, and the elution volume, we estimate peripheral FXP circulating in the blood is approximately six times the value reported in the assay.

Individual FXP expression was reported as a 20% trimmed mean of the observed concentrations between the sample quintuplicates. The sample mean is prone to undue influence by extreme observations, while the sample median is inefficient if the data are normally distributed. The 20% trimmed mean, i.e., the average of the three central values, is used as a compromise between the sample mean and sample median. Differences in protein expression between groups were analyzed using either mixed-effects analysis with Šídák’s multiple comparisons test, Kruskal–Wallis tests with Dunn’s multiple comparison adjustment, unpaired t-tests, or two-tailed Mann–Whitney tests when appropriate in GraphPad Prism software (Version 9.1.0). A p-value < 0.05 was considered significant.

### Assay reproducibility analysis

The stability of FXP over time was analyzed using the Intra-Class Correlation Coefficient (ICC) via SAS® 9.4 (operating on W32_10PRO) software. Specifically, the agreement across time was measured using the ICC where the response, FXP, was modeled as a function of subject identification number and week. The Shrout–Fleiss measure of reliability, ICC, was used where the subjects were evaluated at the various repeated measure timepoints^23^. The ICC here was the same regardless of whether the weeks were treated as a fixed or random variable.

**Figure 2:**
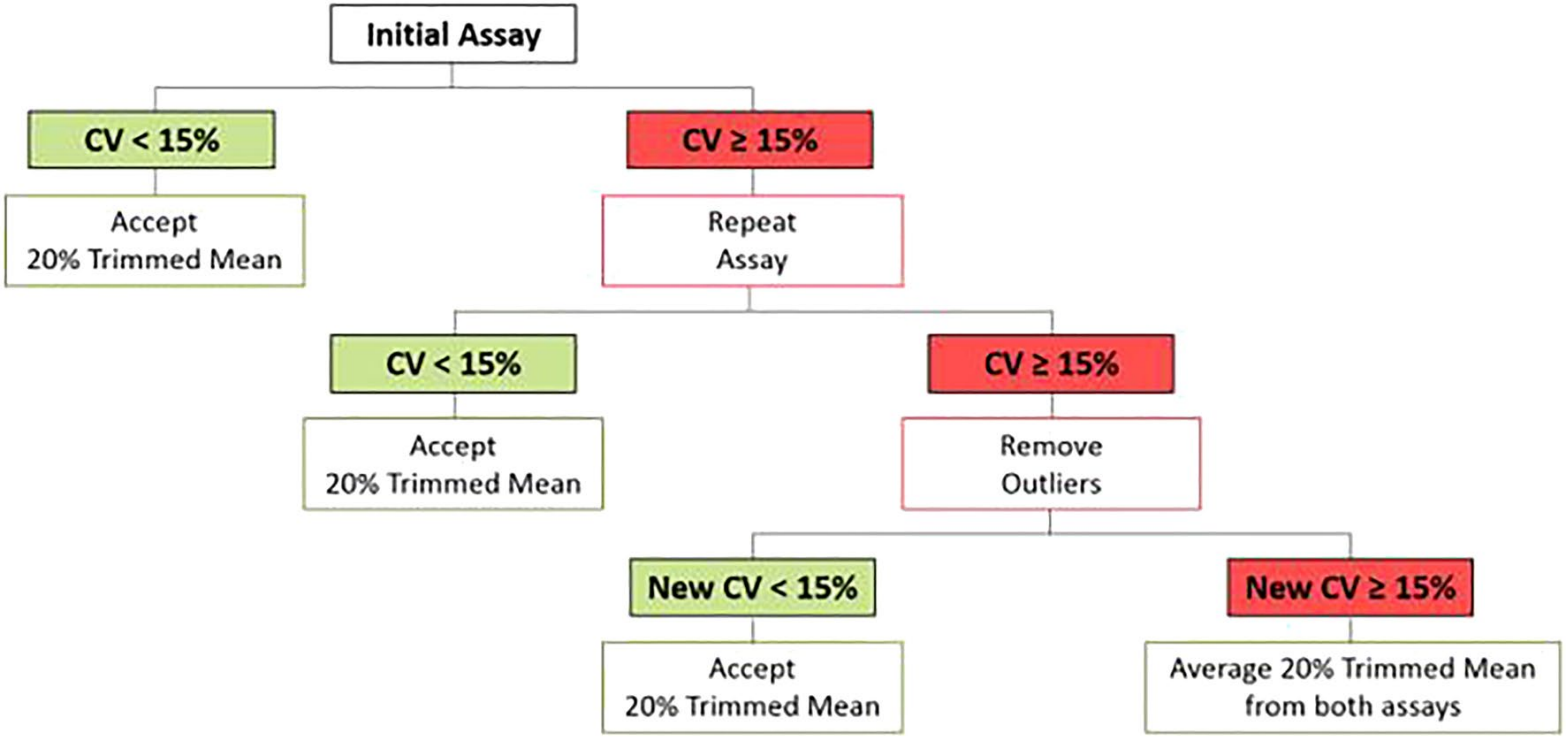
Result acceptance decision tree.

To best characterize the assay, we completed rigorous testing of its performance statistics. We measured intra-assay variability, inter-plate variability, inter-draw variability, and inter-card variability to determine an acceptable standard of variation. Previous studies^23,24^ using the Luminex-based immunoassay platform for FXP-unrelated assays have reported intrinsic variabilities and ranges of acceptable variation through use of the coefficient of variation (CV). Based on those reports, we defined poor variation as CV > 15%, good and acceptable variation as CV < 15%, and excellent variation as CV < 5%. For the calculation of the performance statistics, all interpolated values were considered regardless of their acceptance as a reported value (Fig. 2). Intra-assay variability, or the variability between replicates, was measured and reported as an average %CV for each set of quintuplicates across all participant samples and plates.

### Outlier removal analysis

We used the coefficient of variation (CV) as the standard measure of variability for this assay. Percent CV was determined using the observed concentration calculated via the BioPlex Manager Software [i.e., CV = (standard deviation (of observed mean quintuplicates)/mean (of observed mean quintuplicates)) × 100]. Due to intrinsic variability within any biological test, we set a threshold for acceptable variation within the quintuplicates from one sample as a CV < 15%. This threshold is within the range of the performance statistics of this assay and in alignment with similar methods^23,24^. If upon the initial assay, a sample set of quintuplicates produced an observed concentration CV < 15%, the result was accepted and reported as the 20% trimmed mean of the observed concentration. If the results of the initial assay indicated CV ≥ 15%, the assay was repeated with a new set of 5 DBS from the original DBS card, if possible. If less than 5 DBS were available, the largest amount of available DBS was used. If the results of the repeated assay indicated CV < 15%, the results of the second assay were accepted and reported as the 20% trimmed mean of the observed concentration. If the CV ≥ 15% for both the initial and repeated assays, both sets of quintuplicates were analyzed in the outlier removal program via SAS software.

This final stage of outlier determination was used to retain as much data as possible while not allowing outliers to adversely affect the overall result. Specifically, the generalized extreme Studentized deviate approach was applied to each sample that was based on five DBS^24^. This method allows for the simultaneous detection of multiple outliers, in this case, two. Note that the position of up to two outliers can be found at either or both extremes of the sample. Once the outlier(s) were removed from the data set, a new CV was calculated with the remaining values. If the new CV < 15%, the 20% trimmed mean of the remaining values was accepted and reported. If no outliers were removed or the new CV ≥ 15%, the average of the 20% trimmed means from both assays was calculated and reported (Fig. 2). These samples (very few, see “Results” section) were not removed as we could not exclude that the observed values represent true variability.

### Correlation of Fragile X Protein blood level and intellectual function

In order to assess the potential clinical significance of FXP in blood as measured by our optimized assay, we examined the linear and non-linear relationship between peripheral FXP and intellectual functioning. For this, we examined a subset of participants with FXS (n = 53) who completed the Abbreviated Battery of the Stanford-Binet, Fifth Edition (SB-5^25^) as part of research evaluations (Table 2). SB-5 full-scale IQ standard scores were converted to deviation scores to provide a better estimate of intellectual ability in FXS participants^25,26^.

**Table 2:** Sub-set of participants with FXS included in IQ correlation analysis.

	Male		Female		Total	
	FXS (n = 28)	TDC (n = 15)	FXS (n = 24)	TDC (n = 4)	FXS (n = 52)	TDC (n = 19)
Age	29.9 (8.8)	27.8 (8.3)	21.8 (8.6)*	17.3 (1.3)	26.2 (9.5)	25.6 (8.6)
Full-scale IQ	46.5 (4.0)***	111.3 (13.6)	76.4 (20.0)	96.3 (9.6)	60.3 (20.4)***	108.1 (14.1)
Deviation IQ	32.2 (17.1)***	108.9 (13.0)	77.5 (18.1)	93.6 (8.9)	53.1 (28.7)***	105.7 (13.6)

*FXS* Fragile X Syndrome, *TDC* Typically Developing Controls, *Indicates p < 0.05, ***Indicates p < .0.001.

### Ethics approval and consent to participate

All human experiments described in this publication were approved by the Cincinnati Children’s Hospital Medical Center Institutional Review Board (IRB # 2013-7327). All human subjects when able provided informed consent for all study procedures and all subjects under guardianship (minors or adults) had their guardian consent for subject participation with assent, when possible, obtain from the subject him or herself.

## Results

### Immunoassay optimization: standard curve and lower limit of detection

**Figure 3:**
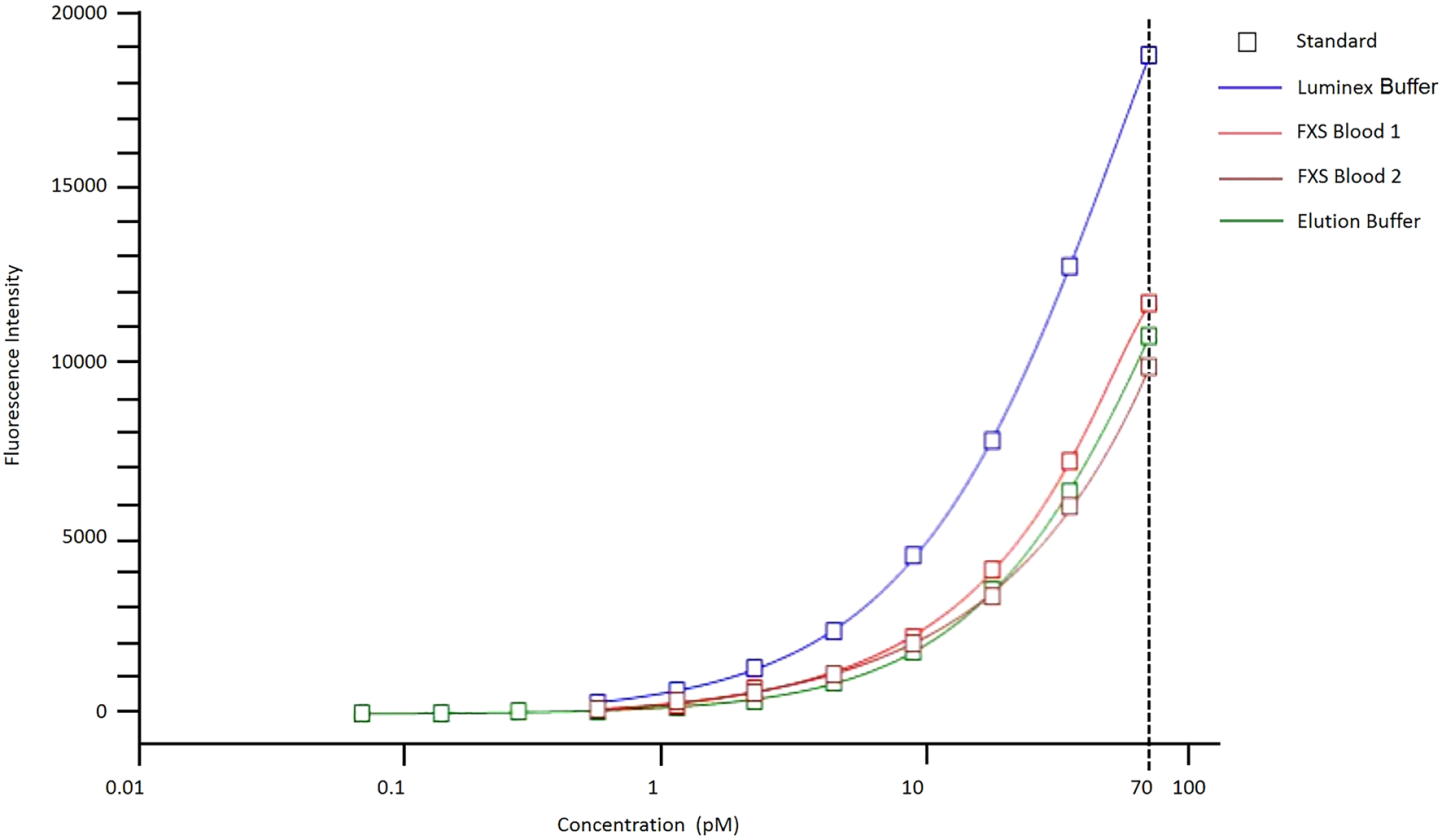
Elution buffer approximates blood matrix effect better than Luminex buffer. Luminex buffer (in blue) was previously used as the standard curve diluent. Due to components within the blood sample demonstrated in the FXS blood curves (both red), fluorescent intensity is globally reduced. This blood matrix effect is mimicked by the elution buffer (in green), allowing the addition of more standard points to lower the detection limit to 0.07 pM. *FXS* Fragile X Syndrome.

Unidentified components in whole blood can cause an overall decrease in signal intensity in fluorescence-based immunoassays, like the Luminex-based immunoassay, termed the blood matrix effect^27–29^. To test if the standard curve conditions used in the Luminex-based FXP assay accounted for the blood matrix effect, we used dried blood spot eluates from two fully methylated FM males as the dilution buffer for the standard protein, GST-SR7. We compared standard curves with this “blood buffer” against the standard curve made with the Luminex buffer previously used^14,18–20^ and a standard curve made with the elution buffer used for sample preparation (Fig. 3). We observed that the blood components globally decreased the median fluorescence intensity at each standard point compared with the respective standard points prepared in Luminex buffer. This finding suggests that previously reported values may have overestimated FXP expression in blood. By contrast, the elution buffer closely mimicked the signal-diminishing effect of the blood matrix. The patient blood curves showed variation in relative intensity, most likely due to minute levels of endogenous FXP. Nevertheless, the elution buffer sufficiently mimicked the signal-diminishing effects of the blood matrix while providing accurate representation of sample preparation and was therefore used in this study.

We then evaluated the other components of the assay to negate any potential complications of interference or background due to the new buffer. Previous optimizations of ELISA-based methods^11,30^ utilized a checkerboard titration system to test multiple antibody conditions on the same test plate. Using this process, we compared different assay conditions to optimize magsphere preparation methods as well as compare two distinct detection antibodies, each at varying concentrations. The largest ratio of median fluorescence intensity between high and low standard points while maintaining a low background signal identified the optimized assay conditions. We determined that using a rabbit anti-human FXP (Abcam, ab17722) provided the highest signal-to-noise ratio with the optimized assay conditions (see Supplementary Table S1 online).

Since the optimized assay conditions decreased the overall background of the assay, we added additional standard points to the low end of the standard curve which decreased the lower limit of detection of the assay. We consistently and reliably have decreased the lower limit of detection to 0.07 pM (Fig. 3). This allows for a more accurate and quantifiable measure for patients with low levels of FXP which had been extrapolated, not quantified, using previous methods.

### Analytical validation of the immunoassay

Intra-assay variability, or the variability between replicates, was measured and reported as an average %CV for each set of quintuplicates across all participant samples and plates. The intra-assay variability could not be calculated for 116 out of 553 (21%) sets of analyzed quintuplicates because the values were outside of the limits of detection and therefore unquantifiable, which was expected since discernable FXP expression would not be expected for all fully methylated FM males. After considering the 21% of unquantifiable quintuplicates, the intra-assay variability was poor for 19% of the quintuplicates while good and acceptable for the remaining 60%. Of the good and acceptable quintuplicates, 47% had excellent variability. The overall average of the intra-assay variability (Table 3) was congruent with similar methods^11,23,24^ and our threshold for outlier removal.

**Table 3:** Performance statistics of the optimized FXP assay.

Variability	Sample size	%CV ± SD
Intra-assay	553 (quintuplicates)	10.1 ± 7.9
Inter-plate	41 (replications)	9.4 ± 10.2
Inter-draw	26 (replications)	6.0 ± 4.1
Inter-card	20 (replications)	2.8 ± 2.3

*CV* Coefficient of variation, *SD* Standard deviation.

To determine the variability between plates (inter-plate variability), blood spots collected on the same DBS card from one participant were analyzed concurrently on two plates. The assays were prepared independently of each other, using separate reagents and randomized analysis order. The inter-plate variability was determined by calculating the CV between the 20% trimmed means from both assay plates. The variability was poor for 15% of replications, and good and acceptable for 70% of replication. The remaining 15% of replications were below the lower limit of detection so therefore the %CV could not be calculated. The overall average inter-plate variability was congruent with the intra-assay variability (Table 3).

Since one K2EDTA collection tube contains enough blood to produce two DBS cards, we determined the inter-card variability, or the variability between both cards from the same blood draw. This value was determined by analyzing each card in quintuplicate on the same assay, prepared with the same conditions applied to each well. Some replications (25%) were below the lower limit of detection and thus not quantifiable, while the remaining 75% samples had good and acceptable variation. This average inter-card variability was lower than the overall variability (Table 3).

### Strategy for outlier removal

The outlier removal analysis was only necessary for 47 quintuplicate samples (out of 527 total sample runs, i.e. 8.9%). Upon completion of the repeated assay, 33 of the samples had acceptable CVs. The remaining 14 repeated samples underwent outlier removal with 9 samples having successful removal of outliers. Only 4 (1 male and 1 female TDC, 2 fully methylated FM males with FXS) samples had a new CV ≥ 15% and thus have reported values of the average 20% trimmed means from both assays.

### Assessing FXP values across diagnostic groups

With the optimized assay conditions, we quantified peripheral FXP in DBS eluate from a total of 187 individuals across the diagnostic categories. Values are reported as a concentration (pM) of FXP in the DBS extract.

**Figure 4:**
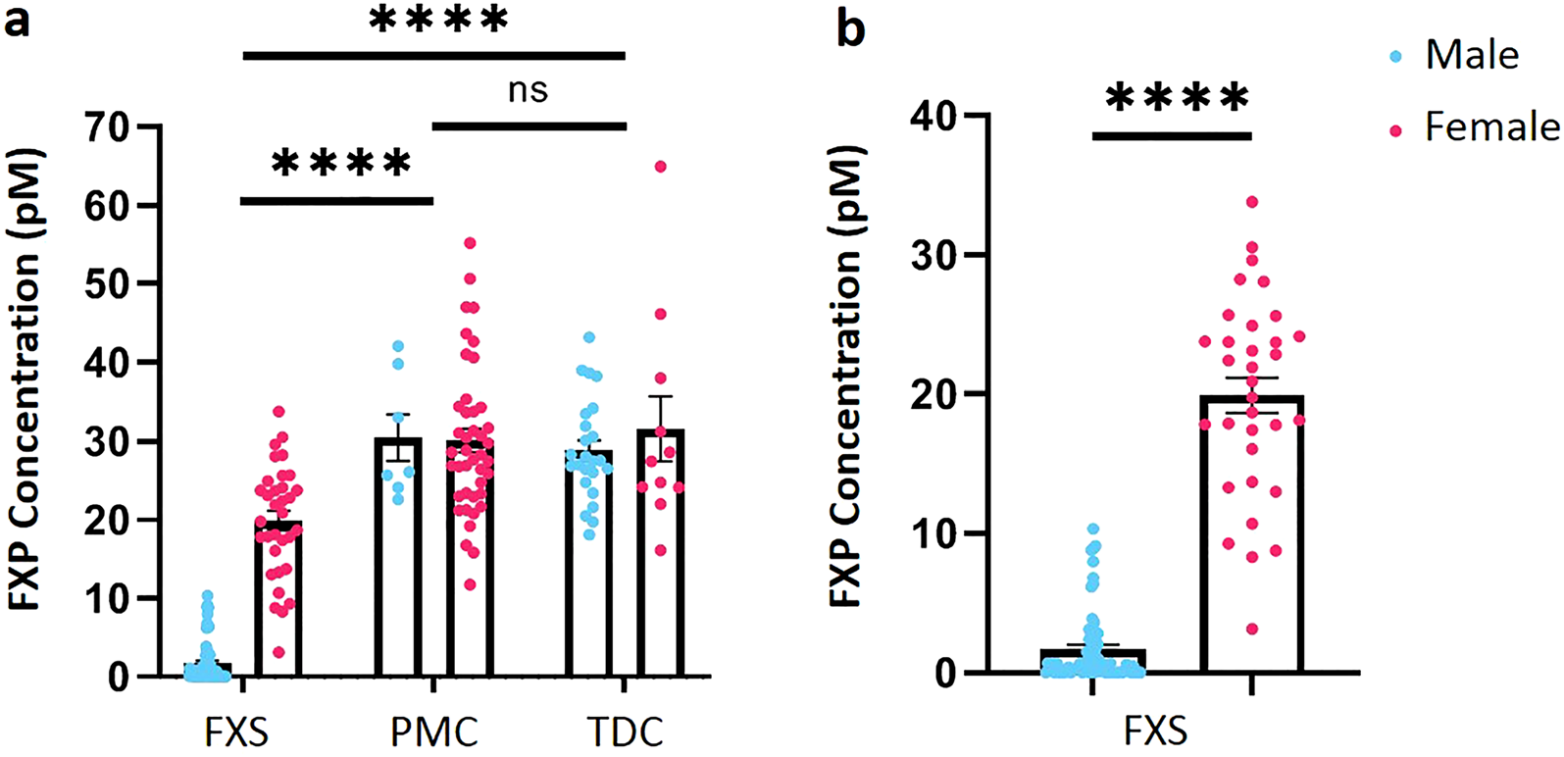
FXP concentration is reduced in peripheral blood of individuals with FXS. (**a**) FXP concentration in all individuals across diagnostic categories from DBS eluate. Graphically males and females are separated into individual bars; however, they were grouped as a diagnostic category when analyzed statistically. FXP concentration is significantly lower in the FXS diagnostic group than PMC and TDC diagnostic groups (Mixed-effects analysis with Šídák's multiple comparisons test; main effect of diagnosis, p < 0.0001 F (1.900, 67.46) = 139.8; main effect of sex p < 0.0001 F (1, 110) = 21.67; interaction diagnosis x sex p < 0.0001, F (2, 71) = 27.30; p(FXS vs PMC) < 0.0001, p(FXS vs TDC) < 0.0001, p(PMC vs TDC) = 0.9882). (**b**) FXP expression is significantly lower in males with FXS than females with FXS (Mann–Whitney *U* = 20, n_1_ = 70 n_2_ = 33, *p* < 0.0001 two-tailed). There were no significant sex differences in FXP expression in PMC (Unpaired T Test, t(47) = 0.1016, *p* = 0.9195 two-tailed) or TDC (Mann–Whitney *U* = 130, n_1_ = 34 n_2_ = 11, *p* = 0.9582 two-tailed). Mean reported with error bars representing SEM. *FXP* Fragile X Protein, *FXS* Fragile X Syndrome, *DBS* Dried Blood Spot, *PMC* Premutation Carriers, *TDC* Typically Developing Controls, *SEM* Standard Error of the Mean, *NS* Not Significant, ****Indicates p < 0.0001.

We first evaluated the expression of FXP between the diagnostic categories. FXP levels are significantly reduced in individuals with FXS when compared with PMCs and TDCs respectively. There was no significant difference in protein concentration between PMCs and TDCs (Fig. 4a). We then analyzed the effects of sex on FXP. There was no significant difference between sex within the PMCs nor within the TDCs (data not shown). However, as expected, there was a significant reduction in FXP in males with FXS in comparison to females with FXS (Fig. 4b).

**Figure 5:**
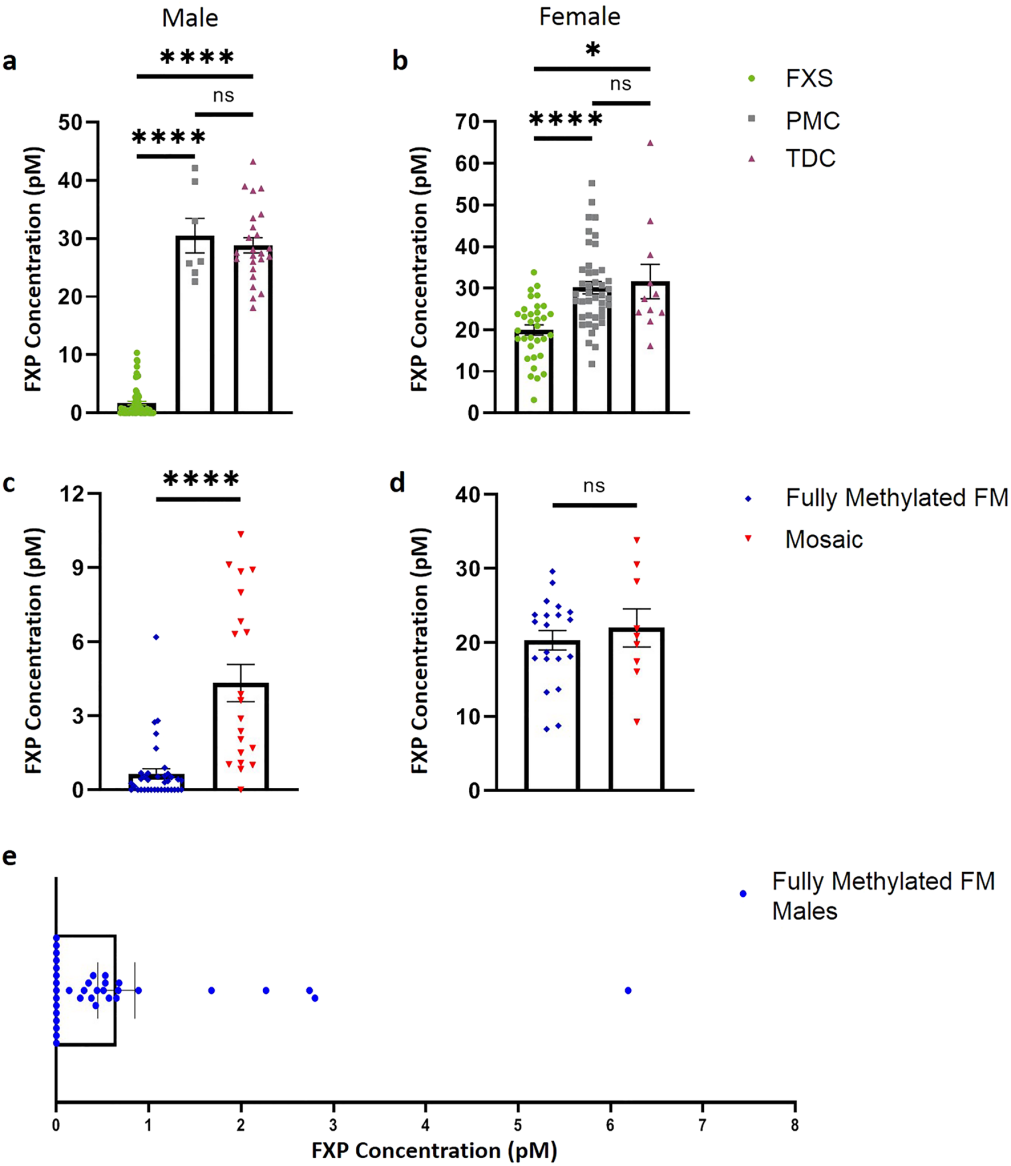
FXP concentration is reduced in males and females with FXS. (**a**) Males with FXS have significantly lower FXP concentrations than PMC and TDC males (Kruskal–Wallis analysis with Dunn’s multiple comparisons; H(3) = 64.40 , *p*(FXS vs PMC) < 0.0001, *p*(FXS vs TDC) < 0.0001). In our sample, there is no significant difference between FXP concentrations in PMC and TDC males (Kruskal–Wallis analysis with Dunn’s multiple comparisons; H(3) = 64.40 , *p* > 0.9999). (**b**) Females with FXS had significantly lower FXP than PMC and TDC females (Kruskal–Wallis analysis with Dunn’s multiple comparisons; H(3) = 22.12, *p(*FXS vs PMC*)* < 0.0001, *p(*FXS vs TDC*)* = 0.0101). There was no significant difference between female PMC and TDC (Kruskal–Wallis analysis with Dunn’s multiple comparisons; H(3) = 22.12, *p* > 0.9999). Note that data shown in (**a**, **b**) are the same as in 4a. (**c**) Fully methylated FM males with FXS have significantly lower FXP concentration than males expressing mosaicism (Mann–Whitney *U* = 68.5, n_1_ = 36 n_2_ = 20, *p* < 0.0001 two-tailed). (**d**) There is no significant difference between fully methylated FM females with FXS and females with methylation mosaicism (Mann–Whitney *U* = 84, n_1_ = 20 n_2_ = 9, *p* = 0.7992 two-tailed). (**e**) Higher resolution of data for fully methylated FM males with FXS illustrates that they express varying levels of FXP, ranging from undetectable to over 6 pM. Mean reported with error bars representing SEM. *FXP* Fragile X Protein, *FXS* Fragile X Syndrome, *DBS* Dried Blood Spot, *PMC* Premutation Carriers, *TDC* Typically Developing Controls, *FM* Full Mutation, *SEM* Standard Error of the Mean, *NS* Not Significant; ****Indicates p < 0.0001; *Indicates p < 0.05.

Due to the nature of X-linked disorders and the fact that females are obligate mosaics, we then compared groups within sexes individually. Males with FXS had significantly lower FXP than their PMC and TDC counterparts. There was no difference between premutation males and TDC males (Fig. 5a). We observed the same trend in the females; females with FXS had significantly lower FXP than PMCs and TDCs, though there was no difference in FXP concentration between female PMCs and female TDCs (Fig. 5b).

Next, we compared males that express the fully methylated FM to their mosaic male counterparts; here, male mosaicism is a grouped category where individuals with either repeat or methylation mosaicism were analyzed as the mosaic group. As expected, fully methylated FM males have significantly lower FXP than mosaic males. Interestingly, we found that there are some fully methylated FM males that consistently express low amounts of FXP, but still less than the average mosaic male FXP level (Fig. 5c,e). Since females are obligate mosaics due to their compensatory X chromosome, we defined mosaicism for females based on methylation status alone. Using this definition, we differentiated between fully methylated FM females and mosaic females that express both full mutation and premutation bands after SB analysis. There was no significant difference between fully methylated FM females and methylation mosaic females (Fig. 5d).

### Validation of assay reproducibility

**Figure 6:**
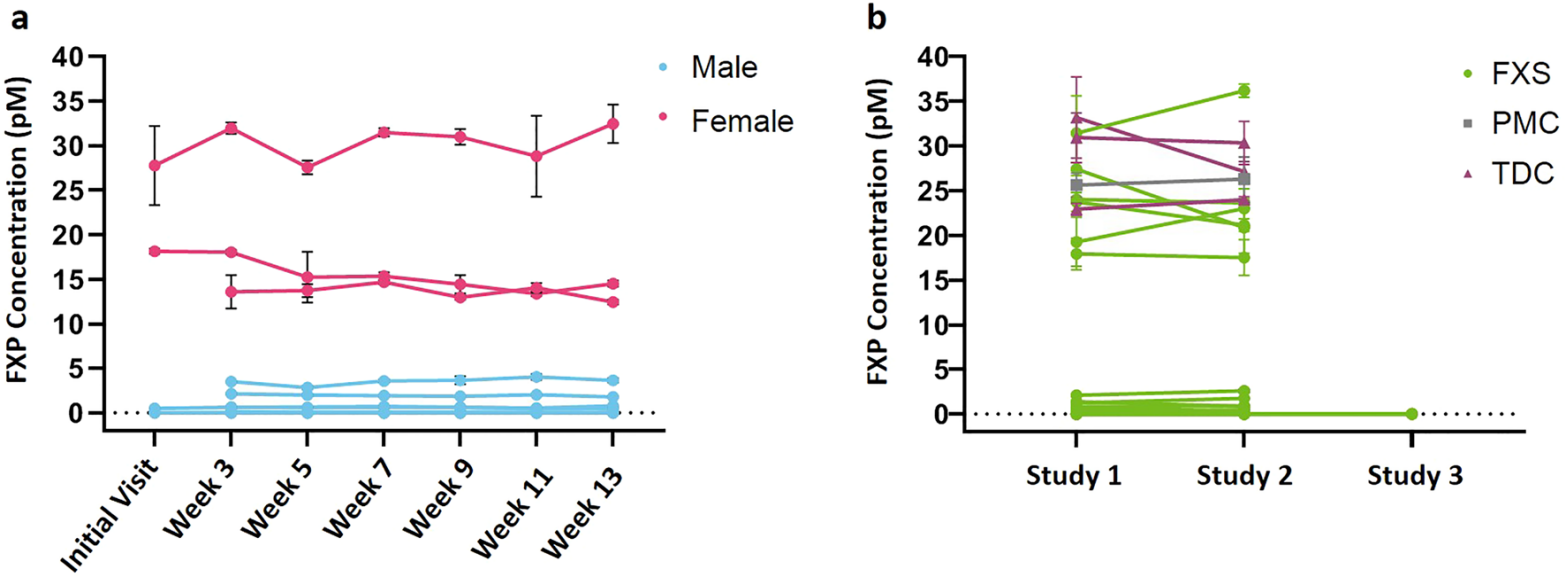
FXP concentrations are consistent over time. (**a**) Repeated bi-weekly FXP levels in 12 adults with FM FXS (9 males; 3 females) showed consistency over 13 weeks (ICC value 98.8), (**b**) Over longer intervals between FXP testing (mean 11 months; range 0.5–30 months), within subject FXP levels showed consistency (ICC value 97.6) in 25 persons (18 males, 7 females; age range 0.5–49.8 years) across all diagnostic categories. Note that study 3 included multiple fully methylated FM males with undetectable FXP levels. *FXP* Fragile X Protein, *FM* Full Mutation, *FXS* Fragile X Syndrome, *ICC* Intra-Class Correlation Coefficient.

We evaluated within subject test–retest reproducibility of blood FXP in two cohorts, a group of 12 adults with FM FXS with FXP levels measured every two weeks totaling seven FXP samples and in 25 persons sampled less frequently (a minimum of two FXP measurements per subject; duration between assays 0.5 to 30 months). In both the short-term (Fig. 6a) and longer-term within subjects repeated FXP testing (Fig. 6b), the FXP results showed excellent intra-individual stability (ICC values were 98.8 and 97.6, respectively). The average variability between two blood draws on different dates of one subject (inter-draw variability) was lower than the overall assay variability (Table 3).

### Correlation of FXP levels with intellectual function

In both males (r_LIN_ = 0.38 p = 0.04) and females (r_LIN_ = 0.53, p = 0.01) with full mutation FXS, higher FXP concentrations were associated with higher Deviation IQ scores. However, when removing six male participants who had either size or methylation mosaicism, this relationship was no longer significant for males with FXS (r = 0.15, p = 0.28). We next examined males with mosaicism and females together, with the thought that because mosaicism confers some production of FXP, the underlying mechanism supporting this relationship with intellectual functioning may be more similar to females with FXS than fully methylated FM males. Deviation IQ and FXP remained significantly related (r_LIN_ = 0.73 p < 0.001).

**Figure 7:**
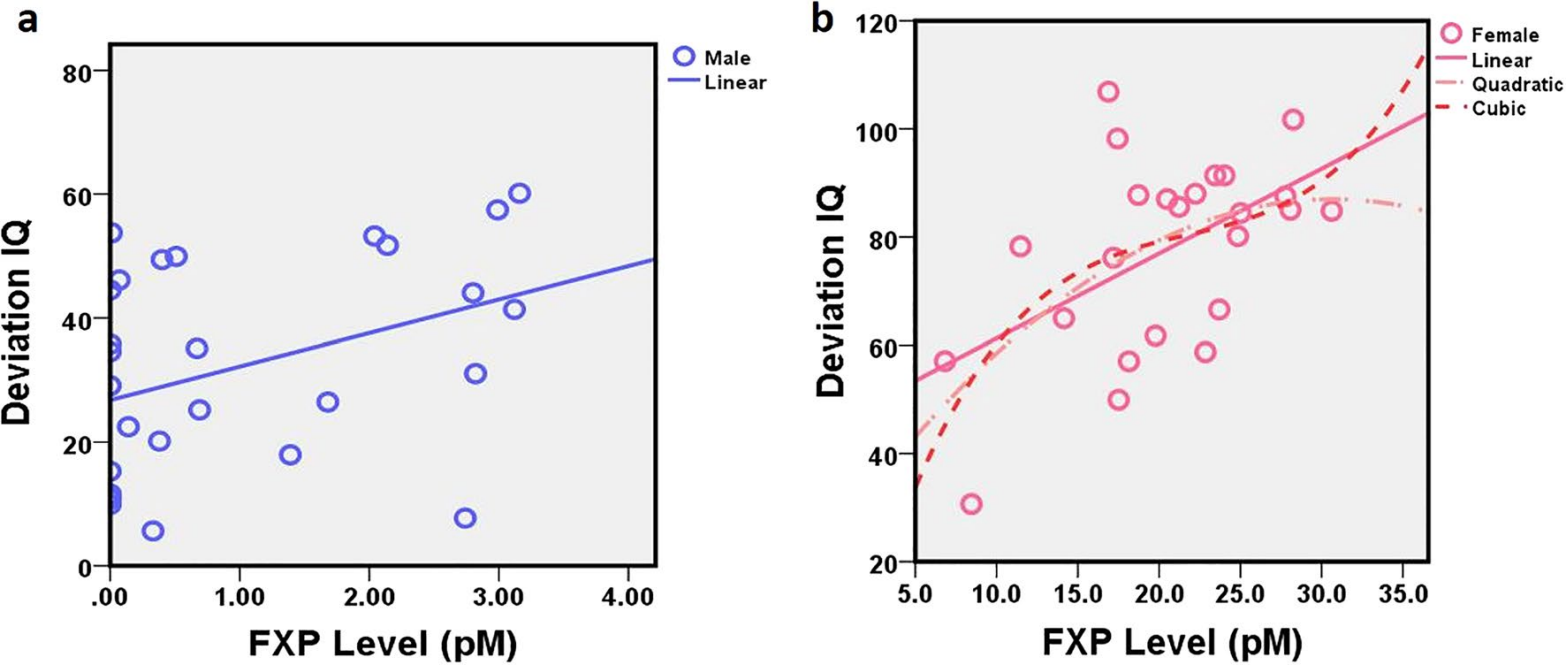
Linear and non-linear correlations between FXP concentrations and Deviation IQ scores for males (**a**) and females (**b**) with FXS. *FXP* Fragile X Protein, *FXS* Fragile X Syndrome.

Previous studies have indicated a non-linear model best describes the relationship between FXP and IQ in FXS^5,6,8,16,17,31–33^. Here, we conducted multiple non-linear models to determine which non-linear function best fit our data separately for males (Fig. 7a) and females (Fig. 7b) with FXS. Among males with FXS, a linear model remained the best fit. In contrast, among females with FXS, logarithmic (r_LOG_ = 0.56, r^2^ = 0.31, p = 0.005) and inverse (r_INV_ = 0.56, r^2^ = 0.31, p = 0.005) functions also fit the data. Next, due to the discrepancy in FXP expression levels between males and females with FXS, we transformed the data with log10 function in order to further assess relationship to IQ with the larger FXS sample. We found significant relationships with linear (r = 0.69, r^2^ = 0.46, p < 0.001), quadratic (r = 0.83, r^2^ = 0.68, p < 0.001), and cubic (r = 0.83, r^2^ = 0.69, p < 0.001) functions.

## Discussion

Given FXS is defined at the core as a protein deficiency disorder, study of FXP is the most directly linked potential protein marker of disease in this field. Thus, the development of a continuous, reliable, and clinically relevant biological marker of fragile X pathophysiology is of critical importance to translational treatment development. Such a marker would have potential clinical utility to predict patient subgroups that may best respond to treatment while also potentially serving as a future moveable biological target of treatment itself. Our current work demonstrates that FXP as measured in peripheral blood by our optimized assay holds promise as a reliable and clinically relevant biological marker in the FXS field.

Previous methods of quantifying FXP have had low signal to noise ratios that increase at the lower limit of quantification and therefore mask potential low level protein expression^20^. Although these previous methods have shown initial clinical relevance by documenting relationships with general cognitive ability, the full scope of its clinical utility is limited due to the restricted range of FXP expression accurately captured by these methods, which used data extrapolation to estimate low levels of FXP^20^. In the current study, using several key assay optimization techniques, including standard curve and reagent optimization, we have developed a reliable and reproducible means to quantify FXP in peripheral blood using DBS cards. Using extensive test–retest strategies we have validated reproducibility of this assay across a wide range of FXP values. In conjunction with lowering the limit of detection to 0.07 pM and our optimizations resulting in increased sensitivity, we identified a new distinct sub-population of clinically defined fully methylated FM males expressing trace or very low levels, but not absent, FXP. This is the first time, to the best of our knowledge, that an FXP assay has reliably and reproducibly documented very low levels of FXP in fully methylated FM subjects in the context of also identifying subjects with no FXP expression. Yet, among fully methylated FM males alone (i.e., excluding males with mosaicism) the relationship between FXP and IQ was no longer significant. This suggests that FXP below a specific threshold may no longer be related to intellectual functioning. Replication and further exploration of this result is needed as is a more comprehensive examination of clinical implications of trace FXP production.

Our study results must be taken in the context of several weaknesses. First, our sample size of PMC and TDC participants was limited. Given this, a thorough interpretation of PMC FXP levels and their clinical relevance will require future work to enroll larger samples of male and female PMCs to better evaluate PMC FXP expression profiles. Though we can differentiate mean FXP differences between persons with FXS and TDC subjects in this small sample, increasing our TDC sample numbers will be important in the future to better define what would be considered a “normal FXP range” in humans. Previous studies used “housekeeping genes”, genomic DNA, or white blood cell counts to normalize FXP values in peripheral blood^20^. Given the widespread effects on protein synthesis by loss of FXP and limited knowledge about the differential expression of FXP across blood cells, here, we chose not to include a normalization step. Future studies are needed to identify the optimal factors for normalization that are not affected by FXS diagnosis. Additionally, we lack at this time data assessing peripheral FXP expression across development in all patient groups evaluated. It will be important to evaluate for potential developmental shifts in FXP expression across developmental windows. Such information will be imperative to interpreting FXP findings and predicting their clinical impact while also enhancing our understanding of FXS pathology thus aiding potential protein-focused therapeutics development in the future.

In addition, our clinical data presented in this manuscript is limited to IQ alone. Comprehensive, multimodal phenotyping of humans with defined FXP blood levels is needed to better understand the potential FXP-brain-behavior relations that may exist in FXS, PMCs, and in the TDC group populations. Although we have demonstrated potential associations between FXP expression and general cognitive function in FXS, we need to use more quantitative and direct evaluations of brain function such as high density electrophysiology, neuroimaging, and additional performance based measures to understand potential relationships between FXP expression and human phenotypes. In particular, it will be imperative to increase our subject sample size in the context of deep phenotyping to determine the clinical relevance, if any, of trace versus absent FXP expression in fully methylated FM males with FXS. Given our FXP analysis is a peripheral tissue assay, clear challenges exist regarding whether a blood finding correlates with true brain FXP variance in humans. Comprehensive neurophysiologic, behavioral, and cognitive phenotyping will play a role in addressing this underlying question as will potential future post-mortem study to evaluate FXP across tissues including brain FXP expression analysis. We remain hopeful that enhanced quantification of brain neurophysiology will in the near term enhance our ability to evaluate the impact of FXP expression as measured in blood to brain activity and function.

Last, to date, we have not evaluated for the potential molecular reasons why we are detecting trace FXP expression in certain males with fully methylated FM FXS. Given the large number of methylation sites on the *FMR1* gene, we hypothesize that regular SB and PCR testing potentially lacks the sensitivity to detect small deviations from true full methylation which could result in some transcription of the *FMR1* gene and resultant FXP production. Future in depth molecular study is warranted to further understand human *FMR1* methylation patterns while also evaluating FXP expression across patient groups in the context of RNA transcript composition and expression. Such future work may be applicable beyond FXS to understand mechanisms of breakthrough protein expression in genes thought to be completely silenced.

## Supplementary Information

Supplementary Table S1.

## Data Availability

The datasets used and/or analyzed during the current study are available from the corresponding author on reasonable request.
