# Supplementary material for: Optimization, validation and initial clinical implications of a Luminex-based immunoassay for the quantification of Fragile X Protein from dried blood spots
Source: Sci Rep. 2022 Apr 4;12:5617. doi: 10.1038/s41598-022-09633-8 (PMC8980090; doi:10.1038/s41598-022-09633-8)
Supplement: Supplementary file 1 — Supplementary Table S1. [file 41598_2022_9633_MOESM1_ESM.pdf]

Detection Antibody	C. A. B. E.	Detection Antibody Concentration											
		1:625			1:1250			1:2500			1:3125		
		**70 pM**	**0.55 pM**	**0 pM**	**70 pM**	**0.55 pM**	**0 pM**	**70 pM**	**0.55 pM**	**0 pM**	**70 pM**	**0.55 pM**	**0 pM**
**R477**	-	12570	409	293	11037.5	280	124	10463	205.5	69	7746	135	45
		12023	489.5	213	11674	331	128	9842.5	184.5	59.5	9181	172	47
	+	12705	413.5	194.5	11698	320	115	9978	214.5	57	9490	169	83
		13805	418	164	13923.5	302.5	138.5	12047	206.5	55	11071.5	159.5	46
		**Detection Antibody Concentration**											
		**1:100**			**1:250**			**1:500**			**1:1000**		
		**70 pM**	**0.55 pM**	**0 pM**	**70 pM**	**0.55 pM**	**0 pM**	**70 pM**	**0.55 pM**	**0 pM**	**70 pM**	**0.55 pM**	**0 pM**
**ab17722**	-	11165	195.5	66	10263	172	50	9598.5	169.5	46	10141.5	154.5	39.5
		11143	218.5	62.5	10905	179.5	53	9396	168	45.5	9482.5	154	39
	+	13806.5	229.5	59	13343	200	46	11804	186	38	**11798**	**177**	**39**
		13324	222	56	12452.5	207.5	45	10781.5	195	44	**11600**	**162**	**37**

Supplementary Table S1. Antibody optimization via the checkerboard titration method. Capture antibody buffer exchange (C.A.B.E.) was optimized due to concerns regarding the efficiency of the bead-antibody coupling reaction.
